# Predicting Long-Term Stability from Short-Term Measurement: Insights from Modeling Degradation in Perovskite Solar Cells during Voltage Scans and Impedance Spectroscopy

**DOI:** 10.1021/acs.jpclett.4c02343

**Published:** 2024-11-15

**Authors:** Will Clarke, Petra Cameron, Giles Richardson

**Affiliations:** † School of Mathematical Sciences, University of Southampton, Southampton SO17 1BJ, U.K.; ‡ Department of Chemistry, University of Bath, Bath BA2 7AY,U.K.

## Abstract

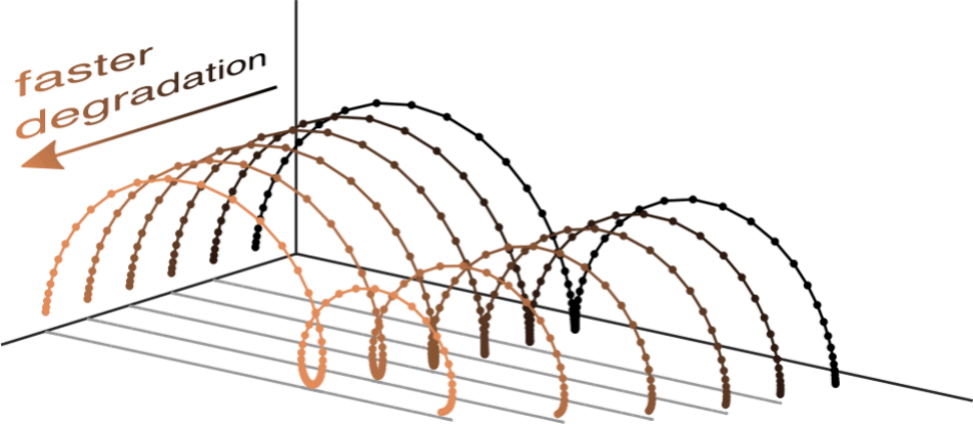

A drift-diffusion model is used to investigate the effect of device degradation on current–voltage and impedance measurements of perovskite solar cells (PSCs). Modifications are made to the open-source drift-diffusion software IonMonger to model degradation via an increasing recombination rate during the course of characterization experiments. Impedance spectroscopy is shown to be a significantly more sensitive measure of degradation than current–voltage curves, reliably detecting a power conversion efficiency drop of as little as 0.06% over a 4 h measurement. Furthermore, we find that fast degradation occurring during impedance spectroscopy can induce loops lying above the axis in the Nyquist plot, the first time this experimentally observed phenomenon has been replicated in a physics-based model.

Over the past decade, perovskites have established themselves as one of the most exciting and promising photovoltaic materials.^7,10,18,22^ Power conversion efficiencies (PCEs) for perovskite solar cells (PSCs) have recently surpassed those of silicon cells, with a current record NREL certified PCE of 26.7%.^20^ All-perovskite and perovskite-silicon tandem devices currently hold impressive record PCEs of 29.1% and 33.9%, respectively.^20^ While these records have solidified perovskites as a significant player in the quest for cheap sustainable energy, there are still challenges that bar their way to large-scale commercial deployment. Although device stability has improved dramatically over recent years,^30^ long-term stability still needs to be improved for commercial development. Additionally, rigorous stability studies are time-consuming, and there is a pressing need for experimental techniques that can infer the long-term degradation trajectory of a device based on sensitive short-term measurements.

While device architectures are often successfully engineered to maximize PCE, this frequently comes at the expense of reduced stability. It is therefore vital to future development that device degradation can be accurately quantified by standard, and rapid, characterization experiments that allow quick assessment of strategies to ameliorate degradation. In this work we consider the effect of degradation on the drift-diffusion model of PSCs. Particular attention is paid to current–voltage curves and impedance spectra. The latter are shown to be a remarkably sensitive tool with which to monitor device stability, based solely on visual inspection of the measured Nyquist plot.

To model the slow degradation of the cell, we introduce a time-dependent prefactor, *r*(*t*), to the bulk Shockley–Reed–Hall (SRH) recombination rate in the perovskite, i.e., eq 64 in ref (4), is replaced by
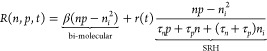
1This prefactor must begin from *r*(0) = 1, representing the “pristine” state of the cell at the beginning of the experiment. For simplicity we consider only the first-order Taylor expansion of the degradation factor,
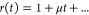
2where μ = *r*′(0) > 0 is the initial rate of degradation. While a linear increase in recombination rate is unrealistic over ultralong time scales, it is a reasonable approximation for the small amount of degradation that occurs during a single experiment. Under this degradation mechanism, the decay in PCE can be simulated via a “perturb and observe” method and a simple empirical formula can be used to relate the degradation rate μ (measured in s^–1^) to the cell’s *T*_90_ time (measured in hours), the time taken for PCE to drop to 90% of its pristine value. This formula is
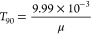
3and is valid for *T*_90_ between 0.15 and 1000 h. This empirical relationship is compared to data from simulated PCE tracking for different values of μ in Figure 1.

**Figure 1:**
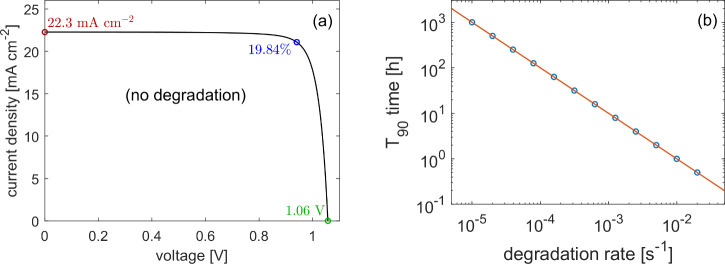
(a) Characterization of the parameter set in the absence of degradation using a slow (1 mV s^–1^) voltage scan. (b) The empirical relationship between degradation rate μ and *T*_90_ time. Circles are from simulated maximum power point tracking using the “perturb and observe” technique and the line is the fit (3).

In a recent work, Thiesbrummel et al.^27^ suggest that degradation in PSC performance occurs predominantly as a result of increased screening of the electric field in the perovskite layer, which, in turn, is a consequence of increases in ion vacancy density, rather than increases in recombination in this layer. However, the type of experimentally observed impedance spectra that we model here cannot be explained solely by increased ion vacancy density as the cell degrades (see Supporting Information (SI), Figure S4), but are associated with an increased recombination rate in the perovskite layer (see Figure 4). This does not necessarily contradict the Thiesbrummel hypothesis, since a rise in vacancy density is often assumed to be accompanied by increases in recombination rate in the perovskite layer due to the role of vacancies as recombination sites.^3,13,24^

Simulations presented in this letter were conducted using a single set of material parameters (listed in SI, Table S1). The parameter set is characterized using a slow current–voltage sweep in the absence of degradation in Figure 1. The pristine PCE of the simulated cell is 19.84% at 1 Sun.

The effects of degradation on a current–voltage sweep are difficult to observe as they are blurred by the hysteretic effect caused by ion migration. Simulating two sweeps, before and after the cell has been held at maximum power point under illumination, however, decouples the two. Comparison between the initial and final *J*–*V* curves can therefore indicate the level of degradation that has occurred during operation. *J*–*V* curves simulated after 4 h of operation under different degradation rates are shown in Figure 2. The scan rate (1 V s^–1^) was chosen as it is below that at which maximum hysteresis is seen (40 V s^–1^) for this parameter set, meaning it gives a reasonable approximation of steady-state behavior while also being fast enough that the increase in recombination rate over the duration of a single scan is negligible. The effect of increased recombination rate through degradation is a reduction in both short-circuit current and open-circuit voltage, along with an increase in the degree of hysteresis observed. Notably, the *J*–*V* curve shows relatively low sensitivity to the increase in recombination. Even after 4 h, only curves corresponding to *T*_90_ ≤ 10 h show significant deviation from the pristine example. This corresponds to *T*_90_ less than 2.5 times the experiment length and a PCE of less than 18.73% after the 4 h period, a drop of at least 1.12% from the pristine value of 19.84% (see Figure 2).

**Figure 2:**
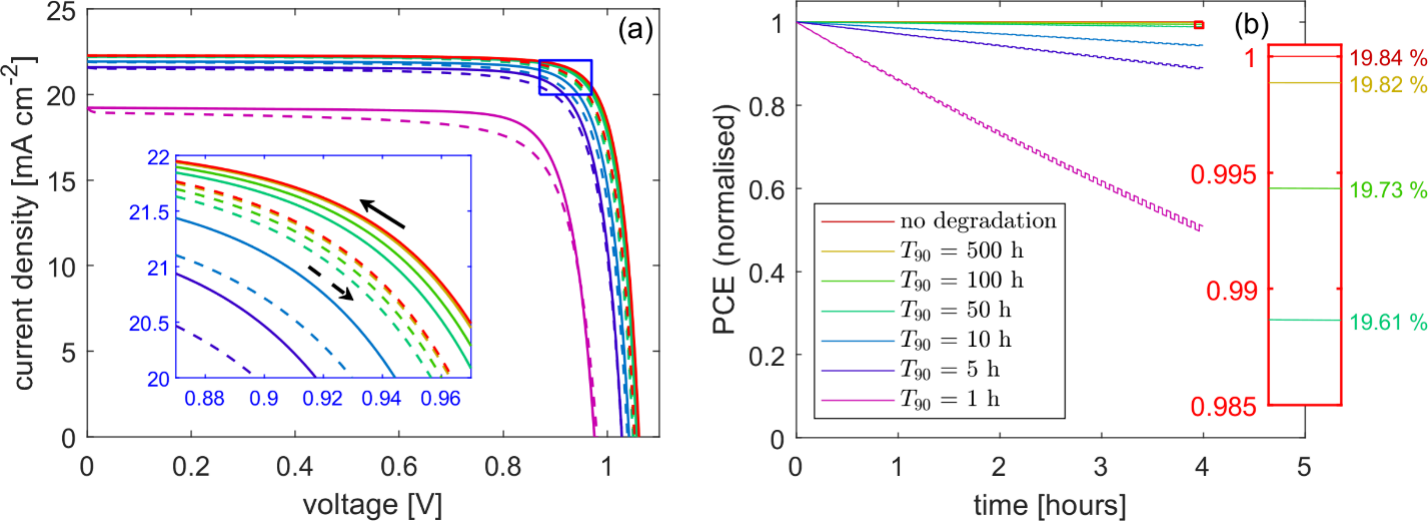
Current–voltage sweeps in the presence of degradation modeled via an increasing bulk SRH recombination rate (1) and computed in IonMonger.^4^ (a) comparison of 1 V s^–1^ sweeps after 4 h of operation against the pristine case in which degradation is eliminated, for a range of degradation rates. Solid lines are the reverse sweep and dashed the forward. (b) Simulated MPP tracking for the same device over the 4 h period of operation, with final PCEs shown in the inset. Parameter set listed in SI, Table S1.

Impedance spectroscopy is another powerful tool used to measure device stability. The effects of degradation on impedance spectroscopy in the model can be grouped into two categories: those that occur between successive measurements and those that occur during a single measurement. We begin with the former. Impedance spectra for the parameter set listed in SI, Table S1 were simulated after the cell had been allowed to degrade for 4 h. For details of the simulation protocol and the computational methods, see the Computational Methods. The results for *T*_90_ between 50 and 1000 h are compared to the pristine spectrum in the absence of degradation in Figure 3. The general shape of the Nyquist plot is reasonably unchanged across these degradation rates, with two clear arcs, both lying above the *x* axis and the radius of the low-frequency arc slightly smaller than that of the high-frequency. The physical origins of these two arcs are well-described in ref (1). The radii of the two arcs, however, exhibit significant variation depending on the rate of degradation. In the context of the RC-RC equivalent circuit,^1,14,28,29^ both the high- and low-frequency resistances decrease as the cell degrades. This follows naturally from the work of Bennett et al.,^1^ in which an RC-RC equivalent circuit was systematically derived from the drift-diffusion equations, identifying both resistances as recombination resistances, inversely proportional to the DC recombination current (*j*_rec_),
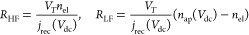
4where *n*_el_ and *n*_ap_ are electronic and apparent ideality factors, respectively, and *V*_*T*_ is the thermal voltage. Here the Nyquist plot consists of two semicircular arcs with diameters given by *R*_HF_ (left arc) and *R*_LF_ (right arc). Notably, an increase in the recombination rate due to degradation corresponds to a reduction in *R*_HF_ and *R*_LF_ and a corresponding decrease in the size of the Nyquist arcs. Accounting for a time-dependent increase in recombination rate, *j*_rec_, *R*_LF_ and *R*_HF_ all become functions of time. By measuring the evolution of the two resistances over time, one can therefore, in principle, reconstruct the rate at which the recombination rate is increasing, and calculate an estimate of long-term stability.

**Figure 3:**
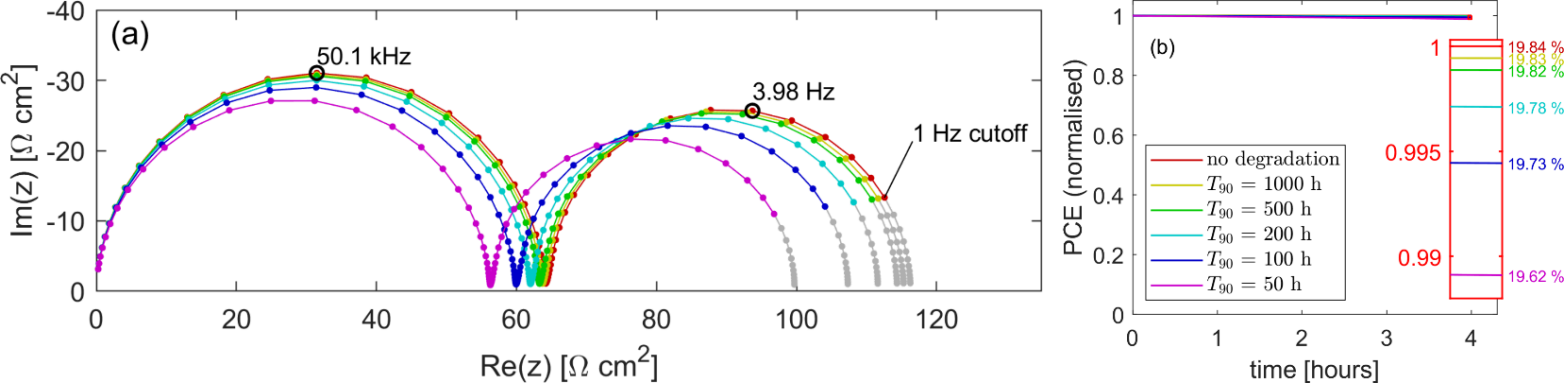
(a) Impedance spectroscopy in the presence of cell degradation via an increasing bulk SRH recombination rate (1) and simulated using Ionmonger.^4^ The *T*_90_ associated with each degradation rate is listed in the legend of (b). Gray data show frequencies that lie below the 1 Hz cutoff that is often used experimentally. Parameter set listed in SI, Table S1. Simulated at *V*_DC_ = 0.9 V under 1 Sun illumination and after a 4 h rest from the pristine state. (b) MPP tracking over the 4 h rest time for the degradation rates used in (a).

These simulated spectra suggest that a cell must show remarkably high stability (*T*_90_ ≥ 500 h) in order for the degradation-induced change in the spectrum to be unnoticeable after 4 h. In this example, the effects of degradation are clearly visible when *T*_90_ = 200 h, corresponding to 50 times the experiment length. At this degradation rate, the PCE drop over 4 h is just 0.06%. In contrast, the simulated *J*–*V* curves discussed above only resolve the effects of degradation over the same 4 h window when *T*_90_ ≤ 10 h (see Figure 2). This demonstrates the high sensitivity of impedance spectroscopy to degradation rates. A PCE decrease of 0.06% is almost imperceptible in a *J*–*V* curve, even in simulations that avoid the limitations of experiment noise, whereas the same degradation is sufficient to very clearly alter the impedance spectrum.

In the simulations described above, the relatively slow rate of degradation has little impact on the cell during the time taken to measure a spectrum, but the cumulative effect over the 4 h rest period is significant. We now turn our attention to faster rates of degradation, such that the cell degrades on the time scale of the measurement, warping the observed spectrum. At such rates, we note that the Nyquist plot will depend on the exact measurement protocol adopted. While we have here used a protocol intended to be representative of common practice in the field, the source code for the version of IonMonger used to compute these figures is freely available as a fork of the IonMonger GitHub repository (https://github.com/WillClarke25/IonMonger-degradation-modelling) in the hope that readers may use it to investigate results for a variety of lab protocols.

Simulated impedance spectra with varying degradation rates are shown in Figure 4. For relatively stable cells (*T*_90_ ≥ 10 h) the degradation that occurs during the experiment time (approximately 8 min) is not enough to significantly alter the spectrum. Toward the end of the experiment, as the lowest frequencies are measured, the increased recombination acts to decrease the observed resistance, observed as a slight drift toward the origin, but the general shape is unchanged. For faster rates of degradation (*T*_90_ ≤ 2 h) the decrease in resistance occurs quickly enough that this drift causes a loop to be formed in the Nyquist plot between the low- and high-frequency arcs. In a stable cell, a plateau typically exists between the high- and low-frequency arcs at which the impedance is real and near-constant. In the presence of degradation, however, the impedance decreases slowly during the measurement of these frequencies, gradually moving toward the origin of the Nyquist plot. The onset of the LF feature therefore closes this loop. Intuitively, the area enclosed by the loop between the low- and high-frequency arcs increases with degradation rate. We note that the minimum degradation rate for which a loop can be observed here corresponds to a *T*_90_ time of 2 h, or approximately 15 times the experiment time. The level of degradation required during an experiment to induce a loop in the Nyquist plot is therefore comparatively small and a loop is observed even though the drop in the cell’s PCE over the 8 min measurement window is only from 19.84% to 19.66%, a difference of 0.18% (see Figure 4b). Once again, by relating the impedance to the recombination current through eq4, eqs 22 and 23 from ref (1)), one can, in principle, calculate an estimate of long-term stability based on observation of a loop in the impedance spectrum. However, parametrizing the decrease in resistance along the path of a single spectrum as a function of time is a significantly more complex challenge than that of comparing multiple spectra measured hours apart. Furthermore, the simulations presented here suggest that a loop is an indicator of poor stability, meaning long-term estimates are of little practical value.

**Figure 4:**
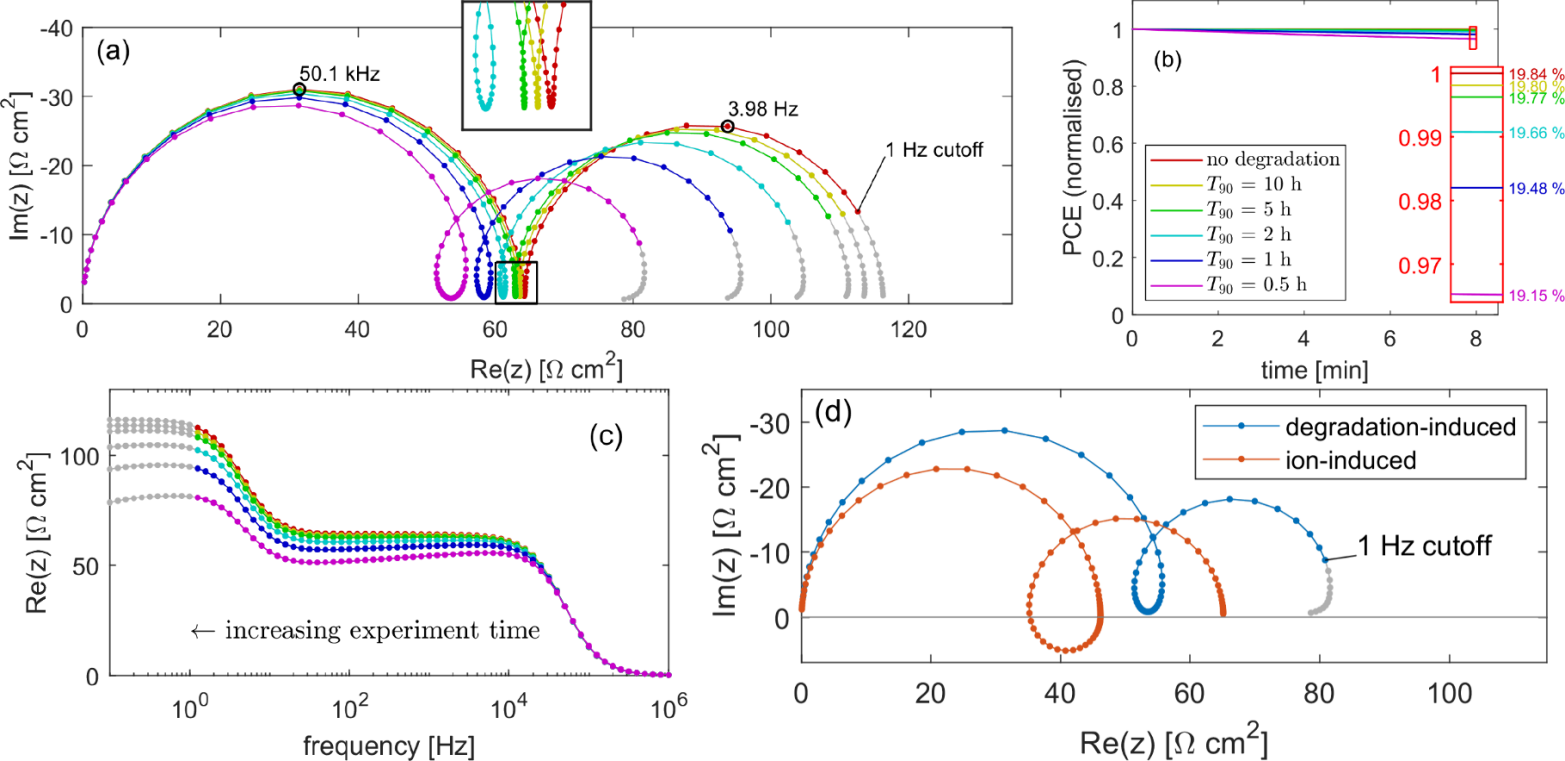
(a) Impedance spectroscopy in the presence of cell degradation modeled via an increasing bulk SRH recombination rate (1) and simulated using Ionmonger.^4^ The *T*_90_ associated with each degradation rate is listed in the legend of (b). Gray data show frequencies that lie below the 1 Hz cutoff that is often used experimentally. Parameter set is listed in SI, Table S1. Simulated at *V*_DC_ = 0.9 V under 1 Sun illumination. (b) MPP tracking for the same degradation rates over the 8 min window taken to measure the impedance spectra shown in (a). The spectra from (a) are shown in (c) with the real part of impedance plotted as a function of frequency. (d) Comparison between loops in simulated Nyquist plots induced by (blue) degradation during measurement and (orange) ion migration coupled with large carrier leakage as described in ref (6). The blue spectrum is taken from (a) with degradation corresponding to *T*_90_ = 30 min. The orange spectrum was simulated using the parameter set “Cell E” in^6^ with no degradation, at *V*_DC_ = 1.0 V under 1 Sun illumination.

In a similar process, the impedance of a perfectly stable cell will tend toward a constant, real value in the low-frequency limit, while that of a cell with degradation will continue to decrease, drifting slowly toward the origin of the Nyquist plot. This can be observed in the gray data in SI, Figure S3. In practice, however, a 1 Hz cutoff frequency is often adopted,^2,8,12,16,17,19,21,23,25,26^ meaning this tail may not be observed. Simulated spectra measuring to frequencies as low as 10 mHz can be found in the SI (Figure S3), showing the slow drift toward the origin.

Loops in the Nyquist plots of impedance spectra of PSCs, occurring between the high- and low-frequency features, were recently shown by Clarke et al.^6^ to be predicted by the standard PSC drift-diffusion model, in the absence of cell degradation, for certain parameter regimes. When one of the perovskite-transport layer band offsets is small, a large density of carriers leaks from the transport layer into the perovskite, and the coupling between the charge carriers introduced into the perovskite and the ion vacancies gives rise to a midfrequency feature in the spectrum. The sign of this feature depends on the majority carrier in question and the dominant recombination pathway. When the MF feature is negative and the LF feature positive, a loop is formed in the Nyquist plot. In Figure 4c, comparison is made between an ion-induced loop predicted by the theory of Clarke et al.^6^ and a degradation-induced loop as described above. This comparison highlights a crucial difference between the two mechanisms: ion-induced loops are caused by a negative MF feature, which leads to a semicircle forming below the axis of the Nyquist plot, whereas degradation cannot change the sign of the impedance response, which means that the spectrum always remains above the axis. These apparently similar Nyquist shapes (which have both been observed experimentally, see e.g.^9,11^) therefore originate from very different phenomena and exhibit characteristic properties which enable them to be distinguished from each other. We have previously shown that ion-induced loops in impedance spectra are linked with inverted hysteresis in the current–voltage curve,^5,6^ whereas degradation-induced loops are only associated with slow deterioration in *J*–*V* curves as in Figure 2.

We have studied the effects of degradation on the drift-diffusion model of a perovskite solar cell by introducing a time-dependent degradation factor to the recombination rate. These simulations are the first of their kind and pave the way for sophisticated physics-based modeling of degradation pathways in PSCs, developing techniques to determine long-term stability based only on short-term experiments. Both current–voltage curves and impedance spectra were simulated using this model, enabling investigation of their relative sensitivities as a measure of degradation. By performing both characterization experiments before and after a 4 h period of operation (and degradation), we show that impedance spectroscopy is far more sensitive to device degradation. Observable changes to the impedance spectrum were detected for *T*_90_ ≤ 200 h, or 50 times the experiment duration, for which the drop in PCE was only 0.06%. In contrast, similar effects were only seen in the current–voltage curve for *T*_90_ ≤ 10 h, or 2.5 times the experiment duration, and the corresponding drop in PCE was 1.12%. This represents a decrease in sensitivity compared to impedance spectroscopy by a factor of approximately 20.

Degradation is observable in the Nyquist plot as a slow drift toward the origin as time progresses. The asymptotic analysis of the drift-diffusion model conducted by Bennett et al.^1^ identified an inverse proportionality between the recombination current at the DC voltage and the radius of the high- and low-frequency arcs in the Nyquist plot, explaining the smooth reduction in |*Z*| as the degradation progresses here. When degradation occurs sufficiently quickly, this effect can induce a loop in the Nyquist plot between the high- and low- frequency features. A drop in the cell’s PCE of as little as 0.18% during the 8 min experiment is sufficient to induce a visible loop, corresponding to a *T*_90_ time of 2 h, or 15 times the experiment duration. Importantly, the degradation-induced loops presented here are easily distinguishable from the ion-induced loops, which were simulated from a drift-diffusion model without degradation by Clarke et al.,^6^ by whether or not they cross the real axis. Building on the findings of Clarke et al.,^6^ this work provides researchers with additional tools to interpret the impedance spectra of PSCs from only a visual inspection of the Nyquist plot, without the need to conduct explicit modeling of each data set.

## Computational Methods

Simulations were performed using the open-source PSC simulation software IonMonger, which solves the drift-diffusion model described in ref (4). The parameter set used here is representative of a typical cell in which the electron transport layer (ETL) is a metal oxide, the perovskite absorber layer (PAL) is a perovskite in which iodide occupies the X site, the hole transport layer (HTL) is a hole-conducting polymer, and light enters through the ETL. The diffusion coefficient and mean density of the mobile ions in the perovskite are set to 3.5 × 10^–10^ cm^2^s^–1^ and 5 × 10^17^ cm^–3^, respectively. These values were obtained from Bayesian parameter estimation conducted by McCallum et al.^15^ for a MAPI-based cell. Recombination is modeled through bimolecular and Shockley-Read-Hall (SRH) recombination in the perovskite bulk and SRH at the material interfaces between the perovskite and the transport layers. At the DC voltage of 0.9 V employed in Figure 4, surface recombination dominates bulk by approximately 1 order of magnitude. The full list of material parameters used for simulations can be found in SI, Table S1. As shown in Figure 1, this parameter set gives a relatively high pristine power conversion efficiency of 19.84%.

In IonMonger, the computational cost of simulating impedance spectra is reduced by utilizing parallel processing.^4^ Once steady state has been achieved at the DC voltage, each frequency measurement is simulated in parallel, beginning from the same initial state. However, this approach is not appropriate when modeling degradation during the experiment, as each frequency measurement must begin from the end state of the preceding measurement. We therefore replace the parallel processing in IonMonger with a simulation protocol in which all measurements are performed sequentially, one after another. This method is validated against the parallel approach in the SI (Figure S1).

**Figure 5:**
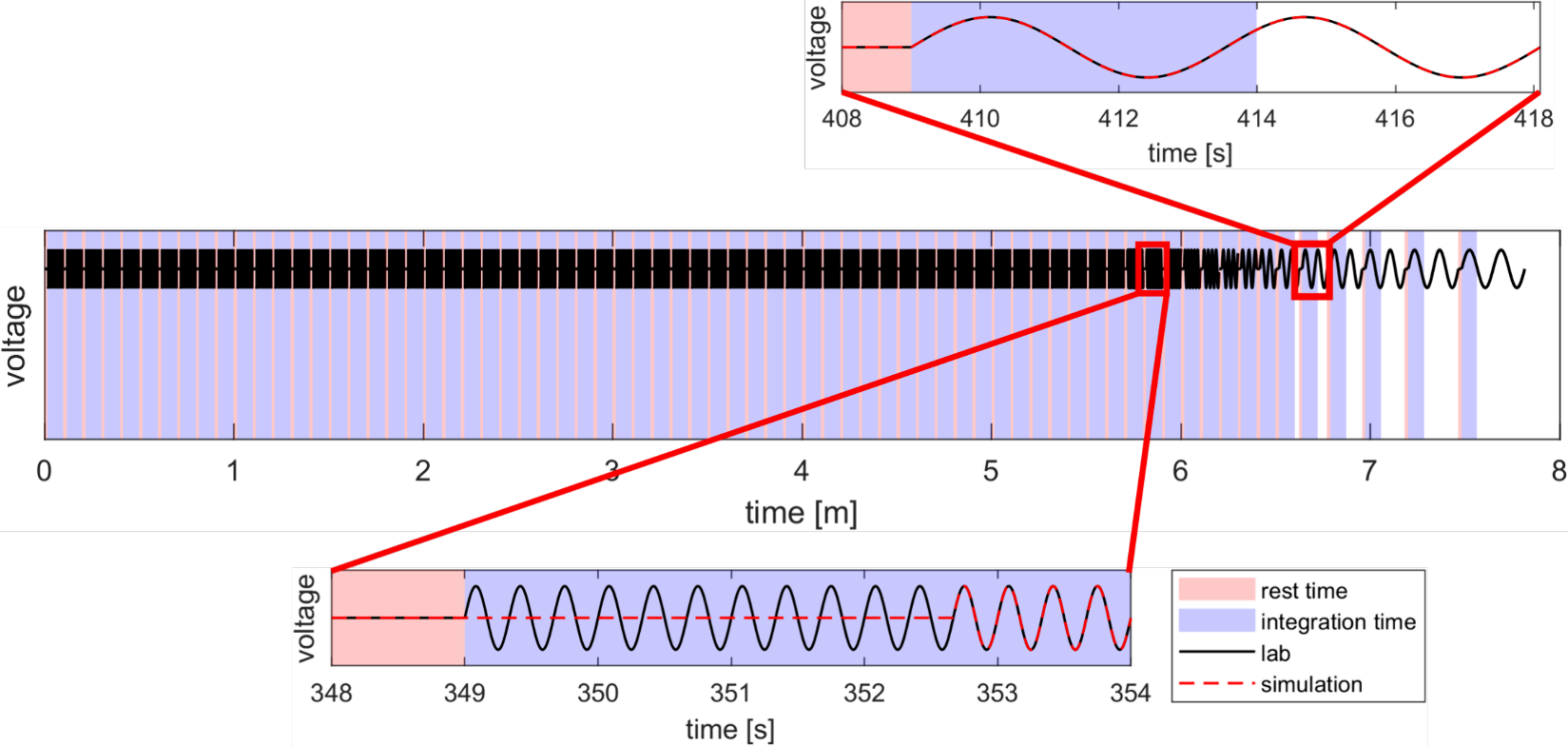
Depiction of the lab protocol for measuring an impedance spectrum. Magnified plots show the simulated protocol used to minimize computation time while occupying the same total experiment time.

While the exact details of experimental impedance protocols vary from lab to lab, we define a representative “lab protocol”, on which to base our simulations, that captures the essence of most impedance experiments. This “lab protocol” is defined by impedance measurements conducted over a range of frequencies beginning with the highest (1 MHz) and ending with the lowest (100 mHz) with a 1 s rest time between each measurement. Frequencies are spaced “ten per decade” between the limits, giving a total of 71. At each frequency the oscillating voltage is applied for a fixed duration, known as the integration time *t*_int_ (set to 5 s). At high frequencies the integration time is sufficient to perform millions of voltage cycles, ensuring high accuracy of the Fourier fit of current response. For frequencies *f* < 2/*T*_int_, two complete voltage cycles are measured, exceeding the integration time to ensure accuracy of the Fourier fit. This lab protocol is depicted in Figure 5 and has a total duration of less than 8 min.

In the case of high frequencies, the number of voltage cycles during the integration time may be in the millions, making fully realistic simulations prohibitively expensive. However, the drift-diffusion model typically only requires a few cycles to reach a quasi-equilibrium, meaning only the final few cycles need to be simulated. In our simulation protocol, computational cost is reduced for high frequency measurements by only simulating the final four voltage cycles following a “dummy” period in which time (and degradation) progresses but the voltage is fixed, as shown in Figure 5. One limitation of IonMonger is that only an integer number of voltage cycles can be simulated. In the frequency range 2/*T*_int_ < *f* < 4/*T*_int_, the lab measurement would take *T*_int_ but the simulation would exceed this. Consequently the simulation protocol for these frequencies consists of a dummy period followed by two full cycles, such that the measurement duration (and thus degradation incurred) is equal to that of the lab protocol. At frequencies *f* < 2/*T*_int_, two full cycles are simulated with no dummy time, as in the lab protocol. This simulation protocol is designed to reduce computation cost while still measuring each value of impedance at the time (and therefore degradation level) as would occur in the lab protocol. This method is validated against simulations in which many more voltage cycles are explicitly modeled in the SI (Figure S2).

The code to solve this updated version of the charge transport model, including sequential impedance measurements, is available as a fork of the IonMonger GitHub repository (https://github.com/WillClarke25/IonMonger-degradation-modelling).

## Supporting Information Available

Validation of sequential vs parallel IS simulation, results for ultralow frequencies, and simulation parameter values (PDF)
