# Supplementary material for: Predicting Long-Term Stability from Short-Term Measurement: Insights from Modeling Degradation in Perovskite Solar Cells during Voltage Scans and Impedance Spectroscopy
Source: J Phys Chem Lett. 2024 Nov 15;15(47):11730–6. doi: 10.1021/acs.jpclett.4c02343 (PMC11613613; doi:10.1021/acs.jpclett.4c02343)
Supplement: Supplementary file 1 — jz4c02343_si_001.pdf [file jz4c02343_si_001.pdf]

# Predicting long-term stability from short-term measurement: insights from modelling degradation in perovskite solar cells during voltage scans and impedance spectroscopy

Will Clarke*[1], Petra Cameron†[2], and Giles Richardson†[1]

[1]School of Mathematical Sciences, University of Southampton, Southampton, UK
[2]Department of Chemistry, University of Bath, UK

November 12, 2024

## Supplementary figures

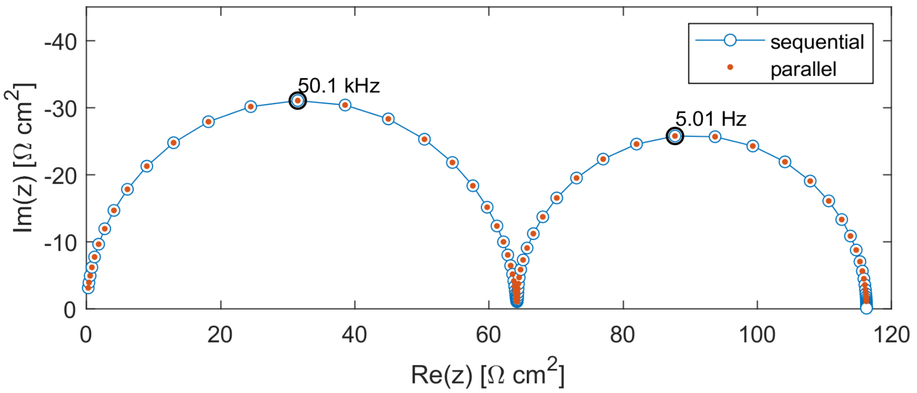


Figure S1: Validation of the sequential simulation method in the absence of degradation. Orange dots show the spectrum computed using IonMonger's standard parallel method [1], and the blue circles the sequential method described in the main text.

*Email address: wc3g16@soton.ac.uk
†Authors contributed equally to this work

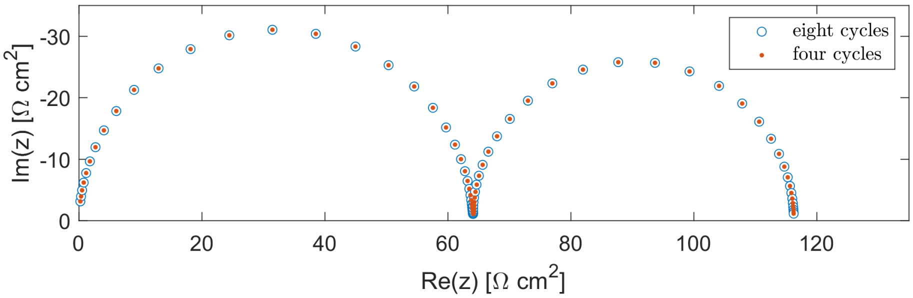


Figure S2: Comparison between simulated impedance for different numbers of simulated cycles. Four full cycles is enough to give a very good approximation of the quasi-steady solution.

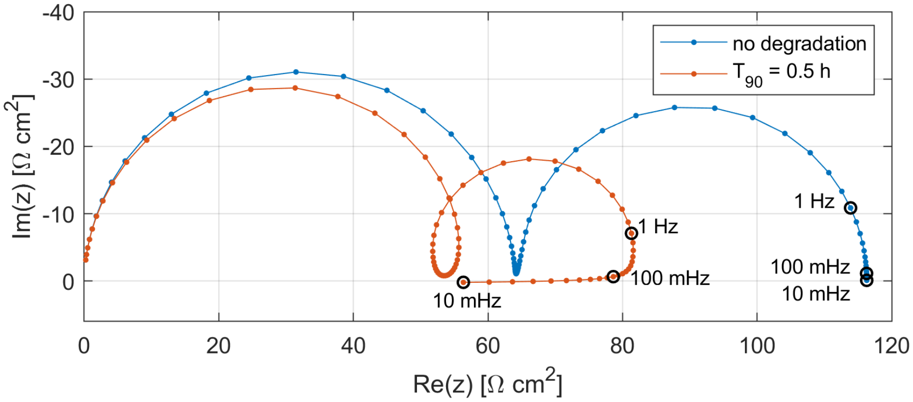


Figure S3: The effect of degradation on low-frequency measurements. Simulation parameters as in Figure 4 with an additional ten measurements to extend the frequency domain to 10 mHz. Total simulated time to perform this experiment is 23 minutes.

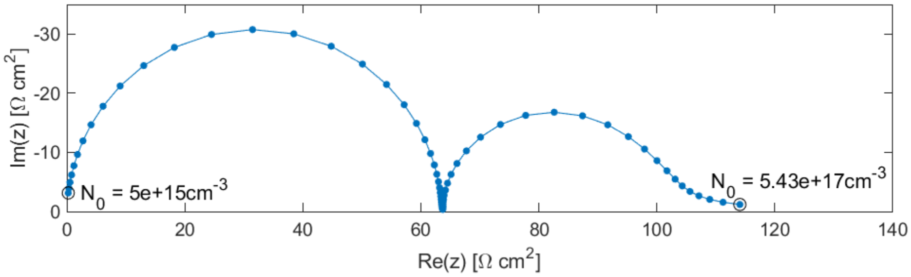


Figure S4: Impedance spectrum from Figure 4 in which degradation is instead modelled through an increasing ion vacancy density, rather than an increasing recombination rate. Each point was simulated using a different value of the mean vacancy density $N_0$ calculated via $N_0(t) = \hat{N}_0 e^{t/\hat{t}}$ where $\hat{N}_0 = 5 \times 10^{15}$ cm$^{-3}$, $\hat{t} = 100$ s, and the time $t$ of each measurement was calculated according to the lab protocol outlined in the Methods section.

| Symbol | Parameter | Value | Unit |
|---|---|---|---|
| $T$ | Temperature | 298 | K |
| $F_{ph}$ | Incident photon flux | $1.4 \times 10^{21}$ | $m^{-2}s^{-1}$ |
| $I$ | Illumination intensity | 1 | Sun equiv. |
| **Perovskite absorber layer (PAL)** | | | |
| $b$ | Layer width | 400 | nm |
| $\varepsilon_p$ | Relative permittivity | 24.1 | $\varepsilon_0$ |
| $\alpha$ | Absorption coefficient | $1.7 \times 10^{7}$ | $m^{-1}$ |
| $E_c$ | Conduction band minimum | -3.7 | eV |
| $E_v$ | Valence band maximum | -5.4 | eV |
| $D_n$ | Electron diffusion coefficient | 1.7 | $cm^2s^{-1}$ |
| $D_p$ | Hole diffusion coefficient | 1.7 | $cm^2s^{-1}$ |
| $g_c$ | Conduction band effective density of states | $8.1 \times 10^{18}$ | $cm^{-3}$ |
| $g_v$ | Valence band effective density of states | $5.8 \times 10^{18}$ | $cm^{-3}$ |
| $N_0$ | Mean ion vacancy density | $5 \times 10^{17}$ | $cm^{-3}$ |
| $D_P$ | Vacancy diffusion coefficient | $3.5 \times 10^{-10}$ | $cm^2s^{-1}$ |
| **Electron transport layer (ETL)** | | | |
| $d_E$ | Effective doping density | $10^{18}$ | $cm^{-3}$ |
| $g_c^E$ | Effective density of states | $5 \times 10^{19}$ | $cm^{-3}$ |
| $E_c^E$ | Conduction band minimum | -4.0 | eV |
| $b_E$ | Layer width | 100 | nm |
| $\varepsilon_E$ | Relative permittivity | 10 | $\varepsilon_0$ |
| $D_E$ | Electron diffusion coefficient | 0.1 | $cm^2s^{-1}$ |
| **Hole transport layer (HTL)** | | | |
| $d_H$ | Effective doping density | $10^{18}$ | $cm^{-3}$ |
| $g_v^H$ | Effective density of states | $5 \times 10^{19}$ | $cm^{-3}$ |
| $E_v^H$ | Valence band maximum | -5.1 | eV |
| $b_H$ | Layer width | 200 | nm |
| $\varepsilon_H$ | Relative permittivity | 3 | $\varepsilon_0$ |
| $D_H$ | Hole diffusion coefficient | 0.01 | $cm^2s^{-1}$ |
| **Recombination parameters** | | | |
| $\beta$ | PAL bulk bimolecular rate | $10^{-9}$ | $cm^3 s^{-1}$ |
| $t_n$ | PAL bulk SRH electron lifetime | 3 | ns |
| $t_p$ | PAL bulk SRH hole lifetime | 300 | ns |
| $v_n^E$ | PAL-ETL surface SRH electron velocity | $10^5$ | $ms^{-1}$ |
| $v_p^E$ | PAL-ETL surface SRH hole velocity | 10 | $ms^{-1}$ |
| $v_n^H$ | PAL-HTL surface SRH electron velocity | 0.1 | $ms^{-1}$ |
| $v_p^H$ | PAL-HTL surface SRH hole velocity | $10^5$ | $ms^{-1}$ |

Table S1: Material parameters used for simulations performed in IonMonger [1]. Note that recombination parameters pertain to the pristine state, *i.e.* $r(t) = 1$.

# References

[1] Will Clarke, Laurence J. Bennett, Yoana Grudeva, Jamie M. Foster, Giles Richardson, and Nicola E. Courtier. Ionmonger 2.0: software for free, fast and versatile simulation of current, voltage and impedance response of planar perovskite solar cells. *Journal of Computational Electronics*, 2022.
